# Predictive performance of lipid parameters in identifying undiagnosed diabetes and prediabetes: a cross-sectional study in eastern China

**DOI:** 10.1186/s12902-022-00984-x

**Published:** 2022-03-24

**Authors:** Yimin Zhou, Guoping Yang, Chen Qu, Jiaping Chen, Yinan Qian, Lei Yuan, Tao Mao, Yan Xu, Xiaoning Li, Shiqi Zhen, Sijun Liu

**Affiliations:** 1 Department of Social Medicine and Health Education, School of Public Health, Nanjing Medical University, 818 Tianyuan East Road, Nanjing, 211166 China; 2 Department of Health Education, Jiangsu Provincial Center for Disease Control and Prevention, 172 Jiangsu Road, Nanjing, 210009 China; 3 Department of Epidemiology, School of Public Health, Nanjing Medical University, 818 Tianyuan East Road, Nanjing, 211166 China

**Keywords:** Lipid, Diabetes, Prediabetes, Predictive

## Abstract

**Background:**

Dyslipidaemia is a risk factor for abnormal blood glucose. However, studies on the predictive values of lipid markers in prediabetes and diabetes simultaneously are limited. This study aimed to assess the associations and predictive abilities of lipid indices and abnormal blood glucose.

**Methods:**

A sample of 7667 participants without diabetes were enrolled in this cross-sectional study conducted in 2016, and all of them were classified as having normal glucose tolerance (NGT), prediabetes or diabetes. Blood glucose, blood pressure and lipid parameters (triglycerides, TG; total cholesterol, TC; high-density lipoprotein cholesterol, HDL-C; low-density lipoprotein cholesterol, LDL-C; non-high-density lipoprotein cholesterol, non-HDL-C; and triglyceride glucose index, TyG) were evaluated or calculated. Logistic regression models were used to analyse the association between lipids and abnormal blood glucose. The area under the curve (AUC) of the receiver operating characteristic (ROC) curve was used to assess the discriminatory power of lipid parameters for detecting prediabetes or diabetes.

**Results:**

After adjustment for potential confounding factors, the TyG was the strongest marker related to abnormal blood glucose compared to other lipid indices, with odds ratios of 2.111 for prediabetes and 5.423 for diabetes. For prediabetes, the AUCs of the TG, TC, HDL-C, LDL-C, TC/HDL-C, TG/HDL-C, non-HDL-C and TyG indices were 0.605, 0.617, 0.481, 0.615, 0.603, 0.590, 0.626 and 0.660, respectively, and the cut-off points were 1.34, 4.59, 1.42, 2.69, 3.39, 1.00, 3.19 and 8.52, respectively. For diabetes, the AUCs of the TG, TC, HDL-C, LDL-C, TC/HDL-C, TG/HDL-C, non-HDL-C and TyG indices were 0.712, 0.679, 0.440, 0.652, 0.686, 0.692, 0.705, and 0.827, respectively, and the cut-off points were 1.35, 4.68, 1.42, 2.61, 3.44, 0.98, 3.13 and 8.80, respectively.

**Conclusions:**

The TyG, TG and non-HDL-C, especially TyG, are accessible biomarkers for screening individuals with undiagnosed diabetes.

**Supplementary Information:**

The online version contains supplementary material available at 10.1186/s12902-022-00984-x.

## Background

Diabetes is a chronic disease that threatens to reduce life expectancy, which affected 463 million adults in 2019 and will affect 700 million worldwide by 2045 [1]. As estimated, the prevalence of diabetes is 9.7% in China [2]. Prediabetes is an intermediate stage from normal glucose tolerance (NGT) to diabetes [3], with the prevalence rising up to 15.5% in China [2]. The situation poses a huge challenge to the financial sustainability of many health care systems around the world, particularly for developing countries [4]. Early detection of abnormal glucose through identifying risk factors [5] might be of great significance to prevent this public health epidemic in China.

Dyslipidaemia is a common feature of insulin resistance and type 2 diabetes and is one of the important risk factors for abnormal blood glucose [6], with a prevalence > 75% among diabetic patients [5]. Dyslipidaemia mainly includes increased levels of triglycerides (TG), small-dense (atherogenic), low-density lipoprotein cholesterol (LDL-C) and decreased levels of high-density lipoprotein cholesterol (HDL-C) [5]. As traditional lipid parameters, total cholesterol (TC), TG, HDL-C and LDL-C are the most commonly used biomarkers to predict diabetes and prediabetes [7–9]. However, nontraditional lipid measures, for instance, non-high-density lipoprotein cholesterol (non-HDL-C) and the ratios between two of the four traditional indicators (TC/HDL-C and TG/HDL-C ratios), were reported to significantly outperform traditional lipid indices in the prediction of abnormal glucose tolerance, mainly because they can provide multiple lipid profiles to make a comprehensive prediction of blood glucose levels [10, 11]. A cross-sectional study in a Chinese community population documented that the TC/HDL-C ratio was superior to traditional lipid indices as a risk marker for diabetes [12]. In recent past years, a simple assessment for metabolic abnormalities, the triglyceride glucose index (TyG) (product of TG and fasting blood glucose), has attracted increasing attention as an excellent marker for the incidence of metabolic diseases because of its good ability to detect insulin sensitivity [13]. A 4-year retrospective longitudinal study indicated that the TyG had discriminative power at a single time point for the diagnosis of diabetes [14]. However, studies on the utility of these lipid parameters to identify diabetes and prediabetes simultaneously are limited.

Therefore, the objectives of the present study were to evaluate the associations between lipid parameters and abnormal blood glucose and to identify the efficacy of lipid predictors in screening for undiagnosed diabetes and prediabetes among community residents in eastern China.

## Methods

### Subjects

Stratified random sampling was performed to select participants from six cities in Jiangsu Province, China, with urban and rural populations included, from March 2016 to June 2016. Details of this cross-sectional study population and procedures have been described elsewhere [15]. A total of 8119 residents were enrolled in the questionnaire survey, anthropometric measurement, and laboratory measurements. Participants were excluded if they had been previously diagnosed with diabetes, were pregnant, or suffered from severe mental disease. The exclusion criteria also included those who did not complete the questionnaire, anthropometric measurement, or blood specimen collection. Finally, a total of 7667 residents were included in the survey. The study protocol was in compliance with the Declaration of Helsinki and approved by the Ethics Review Committee of Jiangsu Provincial Centers for Disease Control and Prevention (No. JSJK2016-B003–03), and informed consent was obtained from all participants before participation.

### Data collection

The standardized questionnaire was used in face-to-face interviews by trained survey personnel to collect sociodemographic information, including gender (men or women), age (years), marital status (married or not), educational attainment, family history of diabetes, history of coronary heart disease, smoking status (current smoking or not) and alcohol drinking status (current drinking or not). The criteria of educational attainment were categorized into below primary school, primary school, middle school and high school and above. A family history of diabetes was defined as at least one family member being diagnosed with diabetes (including parents, siblings and offspring).

Anthropometric indicators, including weight, height, systolic blood pressure (SBP) and diastolic blood pressure (DBP), were measured by trained observers. Weight and height measurements were accurate to 0.01 kg and 0.01 cm, respectively. Blood pressure was measured three times on the right arm of each participant after 5 min of resting in a quiet room, and the mean value was recorded. Body mass index (BMI) was calculated as the weight (kg)/height^2^ (m^2^).

Plasma levels of glucose, including fasting plasma glucose (FPG) and 2-h plasma glucose (2 h PG), TC, TG, HDL-C and LDL-C, were analysed by local hospitals in the county or the Centers for Disease Control and Prevention. Details were previously described [15]. Non-HDL-C was calculated by subtracting HDL-C from TC. The TC/HDL-C and TG/HDL-C ratios were calculated as the ratios of TC (mmol/L) to HDL-C (mmol/L) and TG (mmol/L) to HDL-C (mmol/L), respectively. The equation of the TyG was ln[TG (mg/dL) × FPG (mg/dL)/2] [13].

### Definition of diabetes, prediabetes and NGT

The 1999 World Health Organization (WHO) diagnostic criteria for diabetes and prediabetes were adopted in this study. Diabetes was defined as FPG ≥ 7.0 mmol/L and/or 2 h PG ≥ 11.1 mmol/L. Prediabetes was defined as 6.1 mmol/L ≤ FPG < 7.0 mmol/L or 7.8 mmol/L ≤ 2 h PG < 11.1 mmol/L. NGT was identified as FPG < 6.1 mmol/L and 2 h PG < 7.8 mmol/L.

### Statistical analysis

Continuous variables are presented as quartiles. Differences among groups were compared by utilizing the Kruskal-Wallis H test. Categorical variables are expressed as numbers and percentages and were compared with the chi square test. Logistic regression models (Model 1: without any adjustment, Model 2: adjustment for some potential confounding factors, such as age, sex, marital status, educational level, family history of diabetes, current smoking and current drinking) were used to explore the correlations between glucose status and lipid parameters. The strengths of associations were estimated by odds ratios (ORs) and 95% confidence intervals (CIs). The discriminatory power of lipid parameters to detect prediabetes or diabetes was assessed by the area under the curve (AUC) of the receiver operating characteristic (ROC) curve. The indicator with the largest AUC was considered the best, and the closer the AUC was to 1, the better the prediction will indicate. The sensitivity, specificity, cut-off points, Youden index, and positive and negative predictive values of the lipid indicators were also calculated. All statistical analyses were conducted using the Statistical Package for Social Sciences for Windows version 26.0 (SPSS, Chicago, IL). *P* < 0.05 was considered statistically significant.

## Results

The demographic and clinical characteristics of the study participants in Jiangsu Province are described in Table 1. There were 7667 participants in total, 5884 with NGT, 1283 with prediabetes and 500 with undiagnosed diabetes. Compared to residents with NGT, residents with prediabetes and diabetes were older, more often males, had a lower level of education, were more likely to be married and had higher proportions of a family history of diabetes and current drinking (*P* < 0.05). In addition, patients with prediabetes and diabetes had higher BMI, FPG, 2 h PG, SBP, DBP, TG, TC, LDL-C, TC/HDL-C, TG/HDL-C, non-HDL-C, and TyG and lower HDL-C than those with NGT (*P* < 0.05).

**Table 1:** Demographic and clinical characteristics of study participants in Jiangsu Province

Variables	NGT (*n* = 5884)	Prediabetes (*n* = 1283)	Diabetes (*n* = 500)	*P* values
Age (years)	43 (32,52)	49 (41,56)	51 (44,57)	< 0.001
Male, n (%)	2479 (42.1)	632 (49.3)	256 (51.2)	< 0.001
Married, n (%)	5363 (91.1)	1219 (95.0)	475 (95.0)	< 0.001
Level of education, n (%)				
Below primary school	518 (8.8)	206 (16.1)	58 (11.6)	< 0.001
Primary school	948 (16.1)	278 (21.7)	101 (20.2)	
Middle school	2461 (41.8)	508 (39.6)	232 (46.4)	
High school and above	1957 (33.3)	291 (22.7)	109 (21.8)	
Family history of diabetes (yes, %)	920 (15.6)	249 (19.4)	122 (24.4)	< 0.001
History of coronary heart disease, (yes, %)	25 (0.4)	13 (1.0)	2 (0.4)	0.028
Current smoking (yes, %)	1266 (21.5)	296 (23.1)	129 (25.8)	0.054
Current drinking (yes, %)	1349 (22.9)	384 (29.9)	160 (32.0)	< 0.001
BMI (kg/m^2^)	24.37 (21.99,26.96)	25.88 (23.84,28.65)	27.41 (24.72,29.53)	< 0.001
FPG (mmol/L)	5.18 (4.90,5.50)	5.96 (5.42,6.30)	7.33 (6.71,8.33)	< 0.001
2hPG (mmol/L)	5.65 (4.96,6.37)	8.24 (7.51,9.10)	12.76 (11.31,15.22)	< 0.001
SBP (mmHg)	123.00 (113.00,136.00)	132.00 (121.00,147.00)	138.50 (128.25,152.00)	< 0.001
DBP (mmHg)	78.00 (70.00,85.00)	82.00 (74.00,90.00)	85.00 (79.00,92.00)	< 0.001
TG (mmol/L)	1.13 (0.81,1.69)	1.43 (0.98,2.06)	1.78 (1.26,2.61)	< 0.001
TC (mmol/L)	4.47 (3.95,5.07)	4.84 (4.28,5.46)	5.07 (4.47,5.67)	< 0.001
HDL-C (mmol/L)	1.34 (1.14,1.56)	1.32 (1.13,1.53)	1.29 (1.08,1.48)	< 0.001
LDL-C (mmol/L)	2.45 (2.09,2.94)	2.76 (2.30,3.26)	2.88 (2.39,3.36)	< 0.001
TC/HDL-C	3.30 (2.78,3.93)	3.64 (3.08,4.28)	3.97 (3.35,4.55)	< 0.001
TG/HDL-C	0.84 (0.55,1.36)	1.06 (0.68,1.74)	1.42 (0.88,2.33)	< 0.001
non-HDL-C (mmol/L)	3.08 (2.57,3.69)	3.49 (2.92,4.07)	3.72 (3.19,4.33)	< 0.001
TyG	8.45 (8.11,8.85)	8.82 (8.43,9.18)	9.26 (8.87,9.72)	< 0.001

Results are shown as quartiles unless stated otherwise

*BMI* body mass index, *FPG* fasting plasma glucose, *2hPG* 2-h plasma glucose, *SBP* systolic blood pressure, *DBP* diastolic blood pressure, *TG* triglycerides, *TC* total cholesterol, *HDL-C* high-density lipoprotein cholesterol, *LDL-C* low-density lipoprotein cholesterol, *non-HDL-C* non-high-density lipoprotein cholesterol, *TyG* triglyceride glucose index

The logistic regression analysis of the risk factors for prediabetes and diabetes is presented in Table 2. After adjustment for age, sex, marital status, educational level, family history of diabetes, current smoking and current drinking, TG, TC, LDL-C, TC/HDL-C, TG/HDL-C, non-HDL-C, and TyG had 11.6, 36.5, 30.4, 30.4, 7.6, 41.7, and 111.1% increased risks of prediabetes, respectively. After adjusting for the abovementioned potential confounding factors, the ORs of the TG, TC, LDL-C, TC/HDL-C, TG/HDL-C, non-HDL-C and TyG for the risk of diabetes were 1.221 (95% CI: 1.171–1.274), 1.636 (95% CI: 1.491–1.796), 1.255 (95% CI: 1.107–1.422), 1.575 (95% CI: 1.458–1.703), 1.147 (95% CI: 1.108–1.188), 1.801 (95% CI: 1.639–1.980) and 5.423 (95% CI: 4.658–6.315), respectively.

**Table 2:** Logistic regression analysis of the risk factors for prediabetes and diabetes

Variables	Model	Prediabetes		Diabetes	
		OR (95%CI)	*P*	OR (95%CI)	*P*
TG (mmol/L)	1	1.132 (1.093,1.172)	< 0.001	1.234 (1.185,1.285)	< 0.001
	2	1.116 (1.077,1.157)	< 0.001	1.221 (1.171,1.274)	< 0.001
TC (mmol/L)	1	1.496 (1.404,1.594)	< 0.001	1.790 (1.637,1.956)	< 0.001
	2	1.365 (1.277,1.459)	< 0.001	1.636 (1.491,1.796)	< 0.001
HDL-C (mmol/L)	1	0.906 (0.762,1.076)	0.260	0.525 (0.390,0.708)	< 0.001
	2	0.777 (0.644,0.937)	0.008	0.424 (0.311,0.578)	< 0.001
LDL-C (mmol/L)	1	1.476 (1.362,1.600)	< 0.001	1.469 (1.308,1.651)	< 0.001
	2	1.304 (1.199,1.420)	< 0.001	1.255 (1.107,1.422)	< 0.001
TC/HDL-C	1	1.326 (1.256,1.401)	< 0.001	1.586 (1.475,1.705)	< 0.001
	2	1.304 (1.230,1.382)	< 0.001	1.575 (1.458,1.703)	< 0.001
TG/HDL-C	1	1.081 (1.049,1.114)	< 0.001	1.153 (1.115,1.193)	< 0.001
	2	1.076 (1.044,1.109)	< 0.001	1.147 (1.108,1.188)	< 0.001
non-HDL-C (mmol/L)	1	1.532 (1.436,1.634)	< 0.001	1.928 (1.761,2.110)	< 0.001
	2	1.417 (1.325,1.516)	< 0.001	1.801 (1.639,1.980)	< 0.001
TyG	1	2.264 (2.056,2.493)	< 0.001	5.245 (4.555,6.039)	< 0.001
	2	2.111 (1.907,2.337)	< 0.001	5.423 (4.658,6.315)	< 0.001

Model 1: without any adjustment

Model 2: adjustment for age, sex, marital status, educational level, family history of diabetes, current smoking and current drinking

**Fig. 1:**
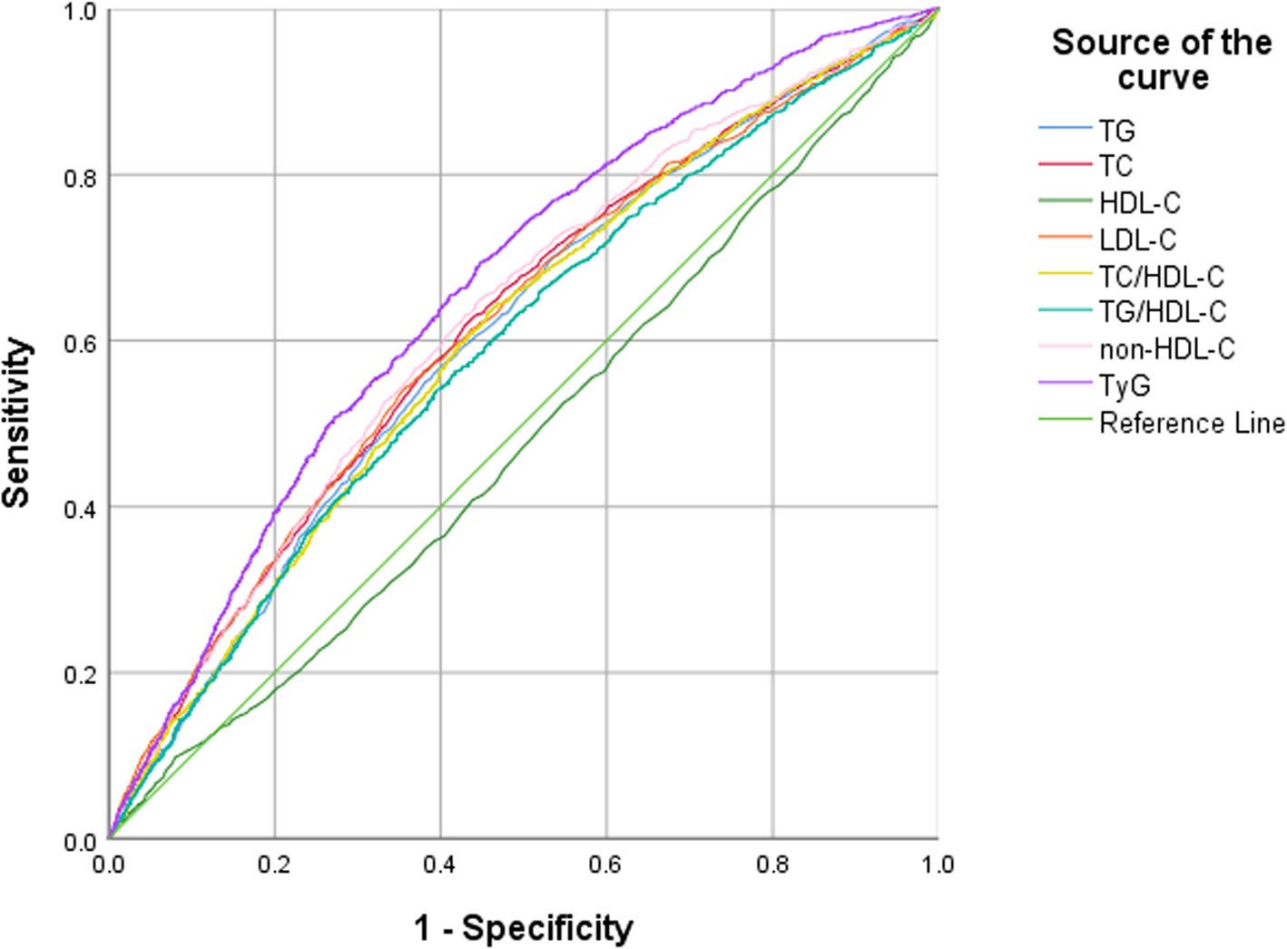
Area under the receiver operating characteristics curves of lipid markers for prediabetes

The predictive values of the lipid parameters on the risk of prediabetes were analysed by ROC curve analysis, and the results are shown in Table 3 and Fig. 1. The AUCs and 95% CIs of the TG, TC, HDL-C, LDL-C, TC/HDL-C, TG/HDL-C, non-HDL-C and TyG for prediabetes were 0.605 (0.593,0.616), 0.617 (0.605,0.628), 0.481 (0.463,0.498), 0.615 (0.604,0.627), 0.603 (0.592,0.615), 0.590 (0.579,0.602), 0.626 (0.614,0.637) and 0.660 (0.649,0.671), respectively. The cut-off points for TG, TC, HDL-C, LDL-C, TC/HDL-C, TG/HDL-C, non-HDL-C and TyG for the prediction of prediabetes were 1.34, 4.59, 1.42, 2.69, 3.39, 1.00, 3.19 and 8.52, respectively.

**Table 3:** Accuracy analysis of different lipid parameters for predicting prediabetes

	AUC (95% CI)	Cut-off points	Sensitivity (%)	Specificity (%)	Youden index	*P* value
TG (mmol/L)	0.605 (0.593,0.616)	1.34	55.03	61.88	0.169	< 0.001
TC (mmol/L)	0.617 (0.605,0.628)	4.59	62.67	56.22	0.189	< 0.001
HDL-C (mmol/L)	0.481 (0.463,0.498)	1.42	63.52	40.45	0.040	0.029
LDL-C (mmol/L)	0.615 (0.604,0.627)	2.69	54.33	64.26	0.186	< 0.001
TC/HDL-C	0.603 (0.592,0.615)	3.39	63.45	54.28	0.177	< 0.001
TG/HDL-C	0.590 (0.579,0.602)	1.00	54.01	60.54	0.146	< 0.001
non-HDL-C (mmol/L)	0.626 (0.614,0.637)	3.19	64.93	55.30	0.202	< 0.001
TyG	0.660 (0.649,0.671)	8.52	69.37	55.27	0.246	< 0.001

*TG* triglycerides, *TC* total cholesterol, *HDL-C* high-density lipoprotein cholesterol, *LDL-C* low-density lipoprotein cholesterol, *non-HDL-C* non-high-density lipoprotein cholesterol, *TyG* triglyceride glucose index

**Fig. 2:**
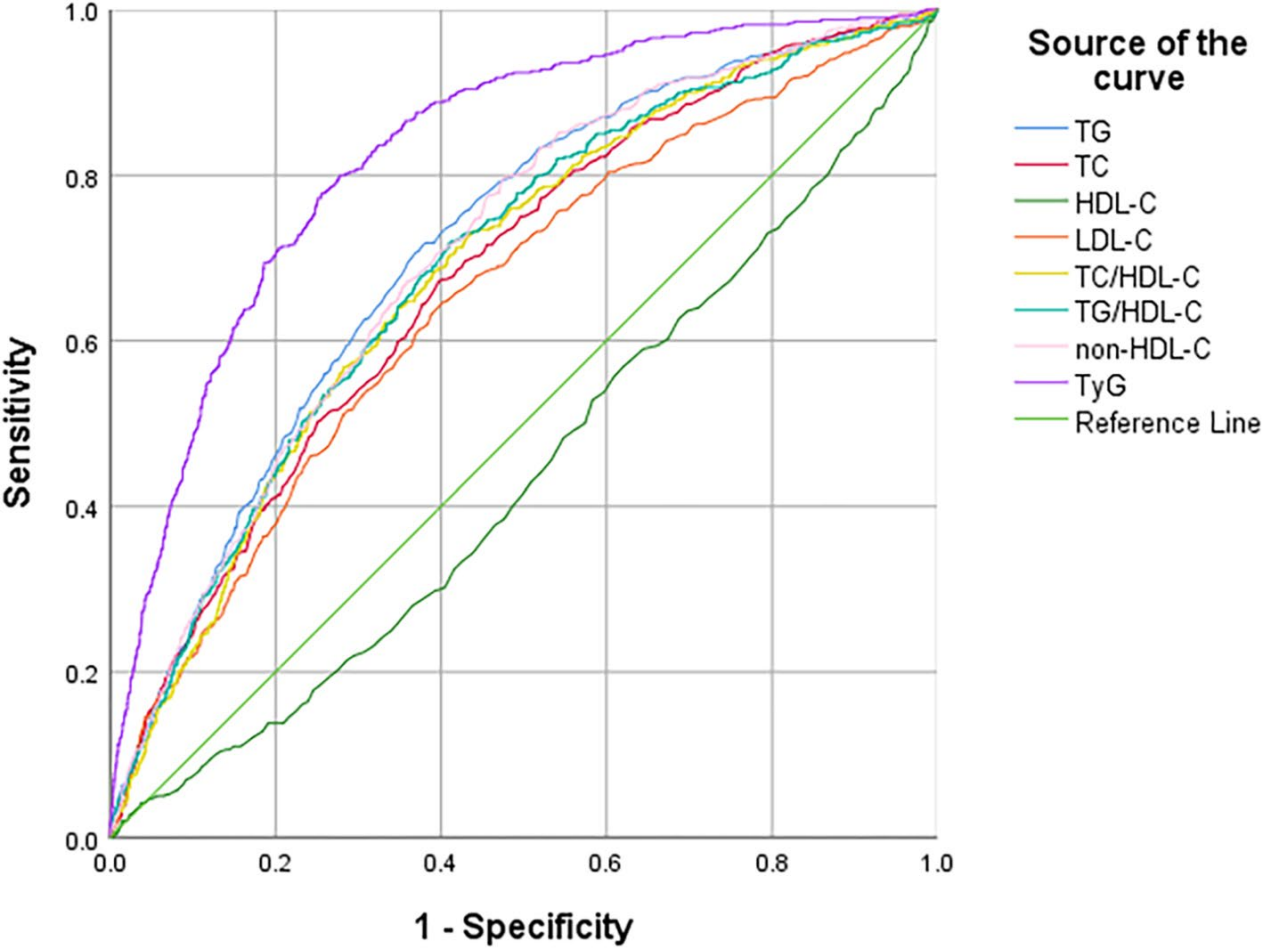
Area under the receiver operating characteristics curves of lipid markers for diabetes

The predictive values of the lipid parameters on the risk of diabetes were analysed by ROC curve analysis, and the results are shown in Table 4 and Fig. 2. The AUCs and 95% CIs of the TG, TC, HDL-C, LDL-C, TC/HDL-C, TG/HDL-C, non-HDL-C and TyG for diabetes were 0.712(0.701,0.723), 0.679(0.667,0.690), 0.440(0.413,0.466), 0.652(0.640,0.663), 0.686(0.674,0.697), 0.692(0.681,0.703), 0.705(0.694,0.716) and 0.827(0.817,0.836), respectively. The cut-off points for TG, TC, HDL-C, LDL-C, TC/HDL-C, TG/HDL-C, non-HDL-C and TyG for the prediction of diabetes were 1.35, 4.68, 1.42, 2.61, 3.44, 0.98, 3.13 and 8.80, respectively.

**Table 4:** Accuracy analysis of different lipid parameters for predicting diabetes

	AUC (95% CI)	Cut-off points	Sensitivity (%)	Specificity (%)	Youden index	*P* value
TG (mmol/L)	0.712 (0.701,0.723)	1.35	71.40	62.34	0.337	< 0.001
TC (mmol/L)	0.679 (0.667,0.690)	4.68	67.20	60.25	0.275	< 0.001
HDL-C (mmol/L)	0.440 (0.413,0.466)	1.42	69.80	40.45	0.103	< 0.001
LDL-C (mmol/L)	0.652 (0.640,0.663)	2.61	64.60	59.79	0.244	< 0.001
TC/HDL-C	0.686 (0.674,0.697)	3.44	72.80	56.73	0.295	< 0.001
TG/HDL-C	0.692 (0.681,0.703)	0.98	71.80	59.06	0.309	< 0.001
non-HDL-C (mmol/L)	0.705 (0.694,0.716)	3.13	79.40	52.62	0.320	< 0.001
TyG	0.827 (0.817,0.836)	8.80	80.00	72.25	0.523	< 0.001

*TG* triglycerides, *TC* total cholesterol, *HDL-C* high-density lipoprotein cholesterol, *LDL-C* low-density lipoprotein cholesterol, *non-HDL-C* non-high-density lipoprotein cholesterol, *TyG* triglyceride glucose index

We also performed stratification analyses for the predictive effect of lipid indicators to discriminate prediabetes and diabetes based on gender, age and BMI (Supplemental Table 1, 2, 3, 4, 5 and 6, Supplemental Fig. 1 and 2). TyG showed the highest diagnostic values for prediabetes and diabetes than other lipid predictors in every group classified by age, gender or BMI.

### Sensitivity analyses

There were 0.4% of NGT (*n* = 25), 1.0% of prediabetes (*n* = 13) and 0.4% of diabetes (n = 2) having coronary heart disease in this study population. When these participants were excluded for the sensitivity analysis, there was no significant change on the discriminatory accuracy and the optimal cut-off values of TG, TC, HDL-C, LDL-C, TC/HDL-C, TG/HDL-C, non-HDL-C and TyG (Supplemental Table 7 and 8).

## Discussion

To direct precise preventive strategies for diabetes and prediabetes, early identification for prediabetes and diabetes using lipid indicators is essential and easier to implement than traditional OGTT in the general population. To the best of our knowledge, the current study is one of the few studies aimed at evaluating the predictive value of lipid parameters for abnormal glycaemic status, including prediabetes and diabetes, in eastern China. In this cross-sectional study, the prevalence of prediabetes and undiagnosed diabetes was 16.7 and 6.8% for residents in Jiangsu Province, respectively. We compared eight lipid parameters (TG, TC, HDL-C, LDL-C, TC/HDL-C, TG/HDL-C, non-HDL-C and TyG) as predictors of prediabetes and undiagnosed diabetes. Among all tested variables, the TyG had the best performance with the highest ORs and the largest AUCs in both diabetic and prediabetic participants. Moreover, the AUCs of TG and non-HDL-C were > 0.7 in the accuracy analysis for predicting diabetes, which indicated that both of them can be potential markers for identifying individuals with diabetes.

Since insulin resistance always occurs before the diagnosis of type 2 diabetes, early screening emerges as an important tool to prevent diabetes. Although the hyperinsulinaemic-euglycaemic clamp test is the gold standard for the determination of insulin resistance [16], it is expensive and cannot be used as a screening test for diabetes in a large population. Other indicators, such as the homeostatic model assessment of insulin resistance (HOMA-IR), were proposed to predict diabetes [17]. Taking into account that laboratories at first-level medical care offices are not able to measure insulin, the TyG is another surrogate index of insulin sensitivity because it is correlated with the HOMA-IR [18] and could be used repeatedly in large-scale observational studies [19]. As a product of TG and FPG, an elevated TyG index means the decreased β-cells and the increased insulin resistance, which may lead to the development of diabetes [20]. A 12-year longitudinal study in Korea reported that a higher TyG significantly predicted type 2 diabetes among middle-aged and elderly people living in communities [21]. A Chinese study indicated that the TyG was a potential predictor to identify individuals at high risk for prediabetes in comparison with other lipid indices [22]. These studies were mainly conducted in diabetic or prediabetic people alone; however, few studies concentrated on the prediction of the TyG for diabetes and prediabetes at the same time. In our study, the screening effects of the TyG to predict the risk of diabetes and prediabetes were analysed simultaneously, and it was found that the TyG was the most efficient index for identifying abnormal blood glucose in all lipid parameters, with an AUC of 0.660 for prediabetes and an AUC of 0.827 for diabetes. Our results are in agreement with the results from a recent study which revealed that the TyG provided good performance to discriminate prediabetes or diabetes in the general German population [23]. This suggested that the TyG, to some extent, can be a promising measurement to screen patients with abnormal blood glucose in different ethnic populations. Additionally, the results seemed that TyG is a better indicator for predicting diabetes compared to prediabetes. This may be related to the fact that insulin sensitivity decreases as blood glucose rises [24]. TyG is an indicator to identify insulin sensitivity [25] and the degree of reduced insulin sensitivity is more pronounced in people with diabetes than in those with prediabetes.

Non-HDL-C, as a nontraditional marker highlighted as an important secondary goal to manage dyslipidaemia [26], has been acknowledged as a potent predictor of cardiovascular disease [27] and a powerful predictor of incident type 2 diabetes in people with NGT [28]. In our study, non-HDL-C was superior to other traditional lipid parameters, including TC, HDL-C and LDL-C, in predicting diabetes and prediabetes. Our results were in concordance with the results of a study in a Canadian population which found a higher non-HDL-C was correlated with the risk of type 2 diabetes and had a better performance than LDL-C and HDL-C in distinguishing between participants with and without diabetes [29]. A case-control study conducted in Han Chinese individuals supported our results, which showed that non-HDL-C is elevated in adults with prediabetes [30]. However, a cohort study previously proposed that non-HDL-C was more informative than traditional cholesterol indices in predicting the risk of diabetes for women but not for men with NGT, which demonstrated that cholesterol levels might be a sex-specific risk factor for diabetes [31]. In addition, our study showed that the AUC of non-HDL-C (0.705) was larger than that of the TG/HDL-C and TC/HDL-C ratios (0.692 and 0.686 respectively) to discriminate diabetes, which was inconsistent with an investigation in Tangshan, China [12]. The possible explanation for the difference may be the various dietary habits in different populations of China.

TG is a traditional component of metabolic syndrome and a cardiovascular risk factor. Plasma levels of TG in lipid profile measurements can also serve as a marker for the prediction of prediabetes in India [32] and in Saudi Arabia [7]. It has been demonstrated that a reduction in TG was associated with decreased diabetes risk in a Norwegian cohort study [33]. High TG provokes lipotoxicity and directly promotes inflammation and endoplasmic reticulum stress, which can lead to insulin resistance [34]. In the present study, TG was a strong predictor of diabetes, with an AUC of 0.712, which was higher than the AUC of the TG/HDL-C ratio (AUC = 0.692). Nevertheless, the predictive utility of TG for diabetes was similar to the TG/HDL-C ratio, with AUCs of 0.580 and 0.577 for TG and TG/HDL-C ratio, respectively, in Iran [35]. Kannel et al. suggested that the TG/HDL-C ratio could be a better predictor of insulin resistance and cardiovascular disease than TG [36], which was contrary to our results. The underlying mechanism for the observed difference is unclear, and we speculate that it may be due to the biological differences between ethnicities. Compared with non-Asians, Asian individuals are more insulin resistant and have lower beta cell function to overcome insulin resistance [37].

The present study has some strengths. First, this is a population-based study with relatively large number of subjects, which could provide high statistical power. Second, this is one of the few studies that compared the lipid index in predicting diabetes and prediabetes simultaneously in eastern China.

Some limitations should be considered for cautious interpretation. First, this is a cross-sectional study that does not allow us to draw any cause-effect relationships. Further cohort studies are required to verify our conclusions in identifying abnormal blood glucose. Second, as we did not measure insulin values in this study, the ratio of insulin-to-fasting glucose across NGT, prediabetes, and diabetes, was not available to correlate insulin levels to triglycerides. Third, variables regarding lifestyle habits such as physical activity, diet behaviour, and medication status of prediabetic participants, which may have potential impacts on the association between lipid indices and diabetes/prediabetes, were not taken into account.

## Conclusions

This is a large population-based cross-sectional study to analyse the predictive values between TG, TC, HDL-C, LDL-C, TC/HDL-C, TG/HDL-C, non-HDL-C, TyG and diabetes/prediabetes in Jiangsu Province. The results showed that the promising values of the TyG, TG and non-HDL-C are accessible biomarkers for screening individuals with undiagnosed diabetes. This study suggests that routine monitoring of lipid parameters to prevent abnormal blood glucose is warranted in residents of eastern China.

## Supplementary Information

**Additional file 1: Supplemental Table 1.** Accuracy analysis of different lipid parameters for predicting prediabetes based on gender.

**Additional file 2: Supplemental Table 2.** Accuracy analysis of different lipid parameters for predicting diabetes based on gender.

**Additional file 3: Supplemental Table 3.** Accuracy analysis of different lipid parameters for predicting prediabetes based on age.

**Additional file 4: Supplemental Table 4.** Accuracy analysis of different lipid parameters for predicting diabetes based on age.

**Additional file 5: Supplemental Table 5.** Accuracy analysis of different lipid parameters for predicting prediabetes based on BMI.

**Additional file 6: Supplemental Table 6.** Accuracy analysis of different lipid parameters for predicting diabetes based on BMI.

**Additional file 7: Supplemental Table 7.** Sensitivity analysis of different lipid parameters for predicting prediabetes of participants with coronary heart disease excluded.

**Additional file 8: Supplemental Table 8.** Sensitivity analysis of different lipid parameters for predicting diabetes of participants with coronary heart disease excluded.

**Additional file 9: Supplemental Figure 1.** ROC curves for predicting prediabetes by lipid index in (a) Men, (b) Women, (c)Age ≥ 46 years, (d) Age < 46 years, (e)BMI ≥ 25.0, and (f) BMI < 25.0.

**Additional file 10: Supplemental Figure 2.** ROC curves for predicting diabetes by lipid index in (g) Men, (h) Women, (i)Age ≥ 46 years, (j) Age < 46 years, (k)BMI ≥ 25.0, and (l) BMI < 25.0.

## Data Availability

The datasets used and/or analysed during this study are available from the corresponding author on reasonable request.

## Abbreviations

NGT: Normal glucose tolerance
OGTT: Oral glucose tolerance test
BMI: Body mass index
FPG: Fasting plasma glucose
2hPG: 2-h plasma glucose
SBP: Systolic blood pressure
DBP: Diastolic blood pressure
TG: Triglycerides
TC: Total cholesterol
HDL-C: High-density lipoprotein cholesterol
LDL-C: Low-density lipoprotein cholesterol
non-HDL-C: non-high-density lipoprotein cholesterol
TyG: Triglyceride glucose index
WHO: World Health Organization
ORs: Odds ratios
CIs: Confidence intervals
AUC: Area under the curve
ROC: Receiver operating characteristics
SPSS: Statistical Package for Social Sciences
