# Supplementary material for: Predictive performance of lipid parameters in identifying undiagnosed diabetes and prediabetes: a cross-sectional study in eastern China
Source: BMC Endocr Disord. 2022 Mar 24;22:76. doi: 10.1186/s12902-022-00984-x (PMC8952267; doi:10.1186/s12902-022-00984-x)
Supplement: Supplementary file 1 — Additional file 1: Supplemental Table 1. Accuracy analysis of different lipid parameters for predicting prediabetes based on gender. [file 12902_2022_984_MOESM1_ESM.docx]

|  | AUC (95% CI) | Cut-off points | Sensitivity (%) | Specificity (%) | Youden index | *P* value |
| --- | --- | --- | --- | --- | --- | --- |
| **Male** |  |  |  |  |  |  |
| TG (mmol/L) | 0.571(0.546,0.595) | 1.34 | 60.13 | 54.01 | 0.141 | <0.001 |
| TC (mmol/L) | 0.622(0.597,0.646) | 4.59 | 64.08 | 53.77 | 0.179 | <0.001 |
| HDL-C (mmol/L) | 0.522(0.496,0.547) | 1.13 | 70.09 | 34.21 | 0.043 | 0.091 |
| LDL-C (mmol/L) | 0.614(0.589,0.639) | 2.55 | 65.65 | 52.68 | 0.182 | <0.001 |
| TC/HDL-C | 0.570(0.546,0.595) | 3.38 | 69.78 | 42.60 | 0.124 | <0.001 |
| TG/HDL-C | 0.551(0.526,0.576) | 1.37 | 44.78 | 65.63 | 0.104 | <0.001 |
| non-HDL-C | 0.618(0.593,0.642) | 3.23 | 67.88 | 51.51 | 0.194 | <0.001 |
| TyG | 0.627(0.603,0.650) | 8.83 | 56.17 | 65.59 | 0.218 | <0.001 |
| **Female** |  |  |  |  |  |  |
| TG (mmol/L) | 0.626(0.603,0.649) | 1.07 | 68.66 | 52.54 | 0.212 | <0.001 |
| TC (mmol/L) | 0.610(0.586,0.633) | 4.60 | 61.14 | 58.38 | 0.195 | <0.001 |
| HDL-C (mmol/L) | 0.457(0.433,0.482) | 1.38 | 54.53 | 53.36 | 0.079 | 0.001 |
| LDL-C (mmol/L) | 0.611(0.587,0.635) | 2.69 | 51.61 | 67.20 | 0.188 | <0.001 |
| TC/HDL-C | 0.621(0.598,0.644) | 3.39 | 58.06 | 62.35 | 0.204 | <0.001 |
| TG/HDL-C | 0.615(0.592,0.639) | 0.81 | 63.29 | 55.62 | 0.189 | <0.001 |
| non-HDL-C | 0.626(0.603,0.649) | 3.19 | 61.14 | 59.56 | 0.207 | <0.001 |
| TyG | 0.682(0.660,0.703) | 8.45 | 72.50 | 56.39 | 0.289 | <0.001 |

TG, triglycerides; TC, total cholesterol; HDL-C, high-density lipoprotein cholesterol; LDL-C, low-density lipoprotein cholesterol; non-HDL-C, non-high-density lipoprotein cholesterol; TyG, triglyceride glucose index.
