# Supplementary material for: Predictive performance of lipid parameters in identifying undiagnosed diabetes and prediabetes: a cross-sectional study in eastern China
Source: BMC Endocr Disord. 2022 Mar 24;22:76. doi: 10.1186/s12902-022-00984-x (PMC8952267; doi:10.1186/s12902-022-00984-x)
Supplement: Supplementary file 2 — Additional file 2: Supplemental Table 2. Accuracy analysis of different lipid parameters for predicting diabetes based on gender. [file 12902_2022_984_MOESM2_ESM.docx]

|  | AUC (95% CI) | Cut-off points | Sensitivity (%) | Specificity (%) | Youden index | *P* value |
| --- | --- | --- | --- | --- | --- | --- |
| **Male** |  |  |  |  |  |  |
| TG (mmol/L) | 0.676(0.644,0.709) | 1.54 | 64.06 | 62.28 | 0.264 | <0.001 |
| TC (mmol/L) | 0.650(0.614,0.685) | 5.06 | 48.83 | 73.78 | 0.226 | <0.001 |
| HDL-C (mmol/L) | 0.470(0.432,0.509) | 1.14 | 42.19 | 64.50 | 0.067 | 0.115 |
| LDL-C (mmol/L) | 0.602(0.564,0.639) | 2.63 | 59.77 | 57.36 | 0.171 | <0.001 |
| TC/HDL-C | 0.635(0.600,0.670) | 3.84 | 60.94 | 61.56 | 0.225 | <0.001 |
| TG/HDL-C | 0.652(0.617,0.686) | 1.17 | 64.84 | 57.64 | 0.225 | <0.001 |
| non-HDL-C | 0.662(0.628,0.696) | 2.99 | 83.59 | 40.42 | 0.240 | <0.001 |
| TyG | 0.799(0.773,0.826) | 8.85 | 78.91 | 66.92 | 0.458 | <0.001 |
| **Female** |  |  |  |  |  |  |
| TG (mmol/L) | 0.735(0.703,0.767) | 1.37 | 70.90 | 68.96 | 0.399 | <0.001 |
| TC (mmol/L) | 0.706(0.676,0.737) | 4.68 | 71.31 | 61.76 | 0.331 | <0.001 |
| HDL-C (mmol/L) | 0.424(0.388,0.460) | 1.42 | 67.21 | 48.05 | 0.153 | <0.001 |
| LDL-C (mmol/L) | 0.697(0.663,0.730) | 2.58 | 70.90 | 60.82 | 0.317 | <0.001 |
| TC/HDL-C | 0.723(0.692,0.755) | 3.45 | 71.31 | 65.46 | 0.368 | <0.001 |
| TG/HDL-C | 0.718(0.684,0.752) | 0.97 | 70.49 | 66.93 | 0.374 | <0.001 |
| non-HDL-C | 0.740(0.711,0.770) | 3.38 | 72.13 | 68.02 | 0.402 | <0.001 |
| TyG | 0.846(0.821,0.872) | 8.80 | 79.10 | 78.21 | 0.573 | <0.001 |

TG, triglycerides; TC, total cholesterol; HDL-C, high-density lipoprotein cholesterol; LDL-C, low-density lipoprotein cholesterol; non-HDL-C, non-high-density lipoprotein cholesterol; TyG, triglyceride glucose index.
