# Supplementary material for: Predictive performance of lipid parameters in identifying undiagnosed diabetes and prediabetes: a cross-sectional study in eastern China
Source: BMC Endocr Disord. 2022 Mar 24;22:76. doi: 10.1186/s12902-022-00984-x (PMC8952267; doi:10.1186/s12902-022-00984-x)
Supplement: Supplementary file 3 — Additional file 3: Supplemental Table 3. Accuracy analysis of different lipid parameters for predicting prediabetes based on age. [file 12902_2022_984_MOESM3_ESM.docx]

|  | AUC (95% CI) | Cut-off points | Sensitivity (%) | Specificity (%) | Youden index | *P* value |
| --- | --- | --- | --- | --- | --- | --- |
| **Age＜46** |  |  |  |  |  |  |
| TG (mmol/L) | 0.635(0.610,0.660) | 1.08 | 69.34 | 51.75 | 0.211 | <0.001 |
| TC (mmol/L) | 0.634(0.608,0.661) | 4.41 | 65.23 | 55.64 | 0.209 | <0.001 |
| HDL-C (mmol/L) | 0.453(0.426,0.480) | 1.41 | 72.22 | 37.60 | 0.098 | 0.001 |
| LDL-C (mmol/L) | 0.643(0.617,0.670) | 2.38 | 69.14 | 52.49 | 0.216 | <0.001 |
| TC/HDL-C | 0.635(0.610,0.661) | 3.40 | 65.43 | 56.93 | 0.224 | <0.001 |
| TG/HDL-C | 0.624(0.598,0.650) | 0.99 | 58.44 | 61.78 | 0.202 | <0.001 |
| non-HDL-C | 0.652(0.626,0.677) | 3.18 | 62.14 | 61.75 | 0.239 | <0.001 |
| TyG | 0.684(0.660,0.708) | 8.44 | 74.07 | 55.03 | 0.291 | <0.001 |
| **Age≥46** |  |  |  |  |  |  |
| TG (mmol/L) | 0.568(0.545,0.592) | 1.46 | 48.81 | 64.04 | 0.129 | <0.001 |
| TC (mmol/L) | 0.570(0.547,0.593) | 4.60 | 66.75 | 45.21 | 0.120 | <0.001 |
| HDL-C (mmol/L) | 0.477(0.454,0.500) | 1.52 | 71.14 | 33.60 | 0.047 | 0.050 |
| LDL-C (mmol/L) | 0.566(0.543,0.590) | 2.69 | 56.84 | 55.48 | 0.123 | <0.001 |
| TC/HDL-C | 0.572(0.549,0.595) | 3.39 | 62.23 | 50.89 | 0.131 | <0.001 |
| TG/HDL-C | 0.561(0.538,0.574) | 1.27 | 38.90 | 71.55 | 0.105 | <0.001 |
| non-HDL-C | 0.579(0.556,0.602) | 3.58 | 48.56 | 64.65 | 0.132 | <0.001 |
| TyG | 0.627(0.605,0.649) | 8.81 | 51.82 | 70.09 | 0.219 | <0.001 |

TG, triglycerides; TC, total cholesterol; HDL-C, high-density lipoprotein cholesterol; LDL-C, low-density lipoprotein cholesterol; non-HDL-C, non-high-density lipoprotein cholesterol; TyG, triglyceride glucose index.
