# Supplementary material for: Predictive performance of lipid parameters in identifying undiagnosed diabetes and prediabetes: a cross-sectional study in eastern China
Source: BMC Endocr Disord. 2022 Mar 24;22:76. doi: 10.1186/s12902-022-00984-x (PMC8952267; doi:10.1186/s12902-022-00984-x)
Supplement: Supplementary file 4 — Additional file 4: Supplemental Table 4. Accuracy analysis of different lipid parameters for predicting diabetes based on age. [file 12902_2022_984_MOESM4_ESM.docx]

|  | AUC (95% CI) | Cut-off points | Sensitivity (%) | Specificity (%) | Youden index | *P* value |
| --- | --- | --- | --- | --- | --- | --- |
| **Age＜46** |  |  |  |  |  |  |
| TG (mmol/L) | 0.766(0.730,0.803) | 1.37 | 78.85 | 66.14 | 0.450 | <0.001 |
| TC (mmol/L) | 0.680(0.637,0.722) | 4.80 | 54.49 | 72.63 | 0.271 | <0.001 |
| HDL-C (mmol/L) | 0.369(0.322,0.415) | 1.20 | 58.33 | 64.24 | 0.226 | <0.001 |
| LDL-C (mmol/L) | 0.621(0.574,0.668) | 2.66 | 50.64 | 69.21 | 0.199 | <0.001 |
| TC/HDL-C | 0.736(0.697,0.775) | 3.87 | 66.03 | 74.21 | 0.402 | <0.001 |
| TG/HDL-C | 0.752(0.713,0.791) | 1.27 | 68.59 | 72.72 | 0.413 | <0.001 |
| non-HDL-C | 0.726(0.688,0.764) | 3.04 | 81.41 | 55.00 | 0.364 | <0.001 |
| TyG | 0.855(0.825,0.885) | 8.99 | 75.00 | 82.72 | 0.577 | <0.001 |
| **Age≥46** |  |  |  |  |  |  |
| TG (mmol/L) | 0.668(0.638,0.698) | 1.34 | 68.60 | 57.47 | 0.261 | <0.001 |
| TC (mmol/L) | 0.634(0.604,0.665) | 4.68 | 71.51 | 49.59 | 0.211 | <0.001 |
| HDL-C (mmol/L) | 0.445(0.413,0.476) | 1.42 | 66.28 | 46.02 | 0.123 | 0.001 |
| LDL-C (mmol/L) | 0.627(0.596,0.658) | 2.62 | 69.48 | 51.46 | 0.209 | <0.001 |
| TC/HDL-C | 0.651(0.621,0.681) | 3.48 | 69.48 | 54.95 | 0.244 | <0.001 |
| TG/HDL-C | 0.656(0.626,0.686) | 0.98 | 68.90 | 56.66 | 0.256 | <0.001 |
| non-HDL-C | 0.661(0.631,0.690) | 3.43 | 67.73 | 57.18 | 0.249 | <0.001 |
| TyG | 0.801(0.777,0.825) | 8.80 | 79.36 | 69.40 | 0.488 | <0.001 |

TG, triglycerides; TC, total cholesterol; HDL-C, high-density lipoprotein cholesterol; LDL-C, low-density lipoprotein cholesterol; non-HDL-C, non-high-density lipoprotein cholesterol; TyG, triglyceride glucose index.
