# Supplementary material for: Predictive performance of lipid parameters in identifying undiagnosed diabetes and prediabetes: a cross-sectional study in eastern China
Source: BMC Endocr Disord. 2022 Mar 24;22:76. doi: 10.1186/s12902-022-00984-x (PMC8952267; doi:10.1186/s12902-022-00984-x)
Supplement: Supplementary file 5 — Additional file 5: Supplemental Table 5. Accuracy analysis of different lipid parameters for predicting prediabetes based on BMI. [file 12902_2022_984_MOESM5_ESM.docx]

|  | AUC (95% CI) | Cut-off points | Sensitivity (%) | Specificity (%) | Youden index | *P* value |
| --- | --- | --- | --- | --- | --- | --- |
| **BMI＜25.0** |  |  |  |  |  |  |
| TG (mmol/L) | 0.596(0.569,0.623) | 1.28 | 46.23 | 70.13 | 0.164 | <0.001 |
| TC (mmol/L) | 0.643(0.618,0.668) | 4.59 | 64.41 | 59.98 | 0.244 | <0.001 |
| HDL-C (mmol/L) | 0.487(0.459,0.515) | 1.84 | 83.17 | 11.87 | 0.050 | 0.350 |
| LDL-C (mmol/L) | 0.626(0.600,0.652) | 2.67 | 52.61 | 68.83 | 0.214 | <0.001 |
| TC/HDL-C | 0.615(0.588,0.641) | 3.40 | 53.97 | 66.32 | 0.203 | <0.001 |
| TG/HDL-C | 0.579(0.551,0.606) | 0.99 | 41.59 | 72.00 | 0.136 | <0.001 |
| non-HDL-C | 0.648(0.623,0.673) | 3.20 | 61.12 | 63.82 | 0.249 | <0.001 |
| TyG | 0.660(0.635,0.685) | 8.53 | 57.83 | 67.23 | 0.251 | <0.001 |
| **BMI≥25.0** |  |  |  |  |  |  |
| TG (mmol/L) | 0.566(0.544,0.589) | 1.50 | 55.74 | 56.20 | 0.119 | <0.001 |
| TC (mmol/L) | 0.584(0.561,0.608) | 4.76 | 54.83 | 58.96 | 0.138 | <0.001 |
| HDL-C (mmol/L) | 0.520(0.497,0.544) | 1.11 | 73.76 | 31.05 | 0.048 | 0.086 |
| LDL-C (mmol/L) | 0.587(0.563,0.610) | 2.75 | 53.66 | 59.81 | 0.135 | <0.001 |
| TC/HDL-C | 0.551(0.528,0.574) | 3.79 | 50.52 | 58.57 | 0.091 | <0.001 |
| TG/HDL-C | 0.548(0.525,0.571) | 1.28 | 48.96 | 59.93 | 0.089 | <0.001 |
| non-HDL-C | 0.581(0.558,0.604) | 3.45 | 54.83 | 57.99 | 0.128 | <0.001 |
| TyG | 0.620(0.599,0.642) | 8.81 | 59.53 | 60.51 | 0.200 | <0.001 |

TG, triglycerides; TC, total cholesterol; HDL-C, high-density lipoprotein cholesterol; LDL-C, low-density lipoprotein cholesterol; non-HDL-C, non-high-density lipoprotein cholesterol; TyG, triglyceride glucose index.
