# Supplementary material for: Predictive performance of lipid parameters in identifying undiagnosed diabetes and prediabetes: a cross-sectional study in eastern China
Source: BMC Endocr Disord. 2022 Mar 24;22:76. doi: 10.1186/s12902-022-00984-x (PMC8952267; doi:10.1186/s12902-022-00984-x)
Supplement: Supplementary file 6 — Additional file 6: Supplemental Table 6. Accuracy analysis of different lipid parameters for predicting diabetes based on BMI. [file 12902_2022_984_MOESM6_ESM.docx]

|  | AUC (95% CI) | Cut-off points | Sensitivity (%) | Specificity (%) | Youden index | *P* value |
| --- | --- | --- | --- | --- | --- | --- |
| **BMI＜25.0** |  |  |  |  |  |  |
| TG (mmol/L) | 0.716(0.671,0.761) | 1.39 | 60.00 | 74.72 | 0.347 | <0.001 |
| TC (mmol/L) | 0.688(0.642,0.733) | 4.68 | 66.43 | 63.82 | 0.303 | <0.001 |
| HDL-C (mmol/L) | 0.476(0.424,0.527) | 1.42 | 58.57 | 50.20 | 0.088 | 0.328 |
| LDL-C (mmol/L) | 0.685(0.639,0.730) | 2.61 | 62.14 | 65.75 | 0.279 | <0.001 |
| TC/HDL-C | 0.660(0.613,0.708) | 3.65 | 50.00 | 76.26 | 0.263 | <0.001 |
| TG/HDL-C | 0.686(0.638,0.734) | 0.99 | 61.43 | 72.24 | 0.337 | <0.001 |
| non-HDL-C | 0.701(0.658,0.745) | 3.04 | 75.00 | 56.57 | 0.316 | <0.001 |
| TyG | 0.840(0.805,0.876) | 8.86 | 72.14 | 84.26 | 0.564 | <0.001 |
| **BMI≥25.0** |  |  |  |  |  |  |
| TG (mmol/L) | 0.653(0.624,0.681) | 1.35 | 75.83 | 48.39 | 0.242 | <0.001 |
| TC (mmol/L) | 0.652(0.623,0.681) | 4.68 | 67.50 | 55.65 | 0.232 | <0.001 |
| HDL-C (mmol/L) | 0.487(0.454,0.519) | 0.94 | 14.44 | 90.01 | 0.045 | 0.407 |
| LDL-C (mmol/L) | 0.606(0.574,0.637) | 2.67 | 62.22 | 55.65 | 0.179 | <0.001 |
| TC/HDL-C | 0.635(0.606,0.664) | 3.83 | 62.22 | 60.05 | 0.223 | <0.001 |
| TG/HDL-C | 0.631(0.601,0.661) | 1.49 | 52.78 | 67.00 | 0.198 | <0.001 |
| non-HDL-C | 0.665(0.637,0.693) | 3.40 | 70.83 | 55.62 | 0.265 | <0.001 |
| TyG | 0.779(0.755,0.803) | 8.99 | 71.67 | 71.98 | 0.436 | <0.001 |

TG, triglycerides; TC, total cholesterol; HDL-C, high-density lipoprotein cholesterol; LDL-C, low-density lipoprotein cholesterol; non-HDL-C, non-high-density lipoprotein cholesterol; TyG, triglyceride glucose index.
