# Supplementary material for: Predictive performance of lipid parameters in identifying undiagnosed diabetes and prediabetes: a cross-sectional study in eastern China
Source: BMC Endocr Disord. 2022 Mar 24;22:76. doi: 10.1186/s12902-022-00984-x (PMC8952267; doi:10.1186/s12902-022-00984-x)
Supplement: Supplementary file 7 — Additional file 7: Supplemental Table 7. Sensitivity analysis of different lipid parameters for predicting prediabetes of participants with coronary heart disease excluded. [file 12902_2022_984_MOESM7_ESM.docx]

|  | AUC (95% CI) | Cut-off points | Sensitivity (%) | Specificity (%) | Youden index | *P* value |
| --- | --- | --- | --- | --- | --- | --- |
| TG (mmol/L) | 0.605(0.588,0.622) | 1.34 | 54.97 | 62.03 | 0.170 | <0.001 |
| TC (mmol/L) | 0.618(0.601,0.635) | 4.59 | 62.85 | 56.31 | 0.192 | <0.001 |
| HDL-C (mmol/L) | 0.480(0.463,0.498) | 1.42 | 63.40 | 40.50 | 0.039 | 0.028 |
| LDL-C (mmol/L) | 0.616(0.599,0.633) | 2.69 | 54.34 | 64.35 | 0.187 | <0.001 |
| TC/HDL-C | 0.604(0.587,0.621) | 3.38 | 64.12 | 53.87 | 0.180 | <0.001 |
| TG/HDL-C | 0.591(0.574,0.608) | 1.00 | 53.94 | 60.68 | 0.146 | <0.001 |
| non-HDL-C | 0.627(0.610,0.644) | 3.19 | 65.00 | 55.42 | 0.204 | <0.001 |
| TyG | 0.661(0.645,0.677) | 8.52 | 69.37 | 55.42 | 0.248 | <0.001 |

TG, triglycerides; TC, total cholesterol; HDL-C, high-density lipoprotein cholesterol; LDL-C, low-density lipoprotein cholesterol; non-HDL-C, non-high-density lipoprotein cholesterol; TyG, triglyceride glucose index.
