# Supplementary material for: Predictive performance of lipid parameters in identifying undiagnosed diabetes and prediabetes: a cross-sectional study in eastern China
Source: BMC Endocr Disord. 2022 Mar 24;22:76. doi: 10.1186/s12902-022-00984-x (PMC8952267; doi:10.1186/s12902-022-00984-x)
Supplement: Supplementary file 8 — Additional file 8: Supplemental Table 8. Sensitivity analysis of different lipid parameters for predicting diabetes of participants with coronary heart disease excluded. [file 12902_2022_984_MOESM8_ESM.docx]

|  | AUC (95% CI) | Cut-off points | Sensitivity (%) | Specificity (%) | Youden index | *P* value |
| --- | --- | --- | --- | --- | --- | --- |
| TG (mmol/L) | 0.715(0.693,0.738) | 1.37 | 71.11 | 63.27 | 0.344 | <0.001 |
| TC (mmol/L) | 0.681(0.657,0.705) | 4.68 | 67.47 | 60.32 | 0.278 | <0.001 |
| HDL-C (mmol/L) | 0.439(0.413,0.466) | 1.42 | 69.49 | 40.50 | 0.100 | <0.001 |
| LDL-C (mmol/L) | 0.653(0.627,0.678) | 2.61 | 64.65 | 59.87 | 0.245 | <0.001 |
| TC/HDL-C | 0.688(0.664,0.711) | 3.44 | 72.93 | 56.87 | 0.298 | <0.001 |
| TG/HDL-C | 0.695(0.671,0.718) | 0.98 | 72.12 | 59.22 | 0.313 | <0.001 |
| non-HDL-C | 0.707(0.685,0.730) | 3.13 | 79.60 | 52.73 | 0.323 | <0.001 |
| TyG | 0.829(0.812,0.847) | 8.80 | 80.40 | 72.37 | 0.528 | <0.001 |

TG, triglycerides; TC, total cholesterol; HDL-C, high-density lipoprotein cholesterol; LDL-C, low-density lipoprotein cholesterol; non-HDL-C, non-high-density lipoprotein cholesterol; TyG, triglyceride glucose index.
