# Supplementary figures and images for: Predictive performance of lipid parameters in identifying undiagnosed diabetes and prediabetes: a cross-sectional study in eastern China
Source: BMC Endocr Disord. 2022 Mar 24;22:76. doi: 10.1186/s12902-022-00984-x (PMC8952267; doi:10.1186/s12902-022-00984-x)

**
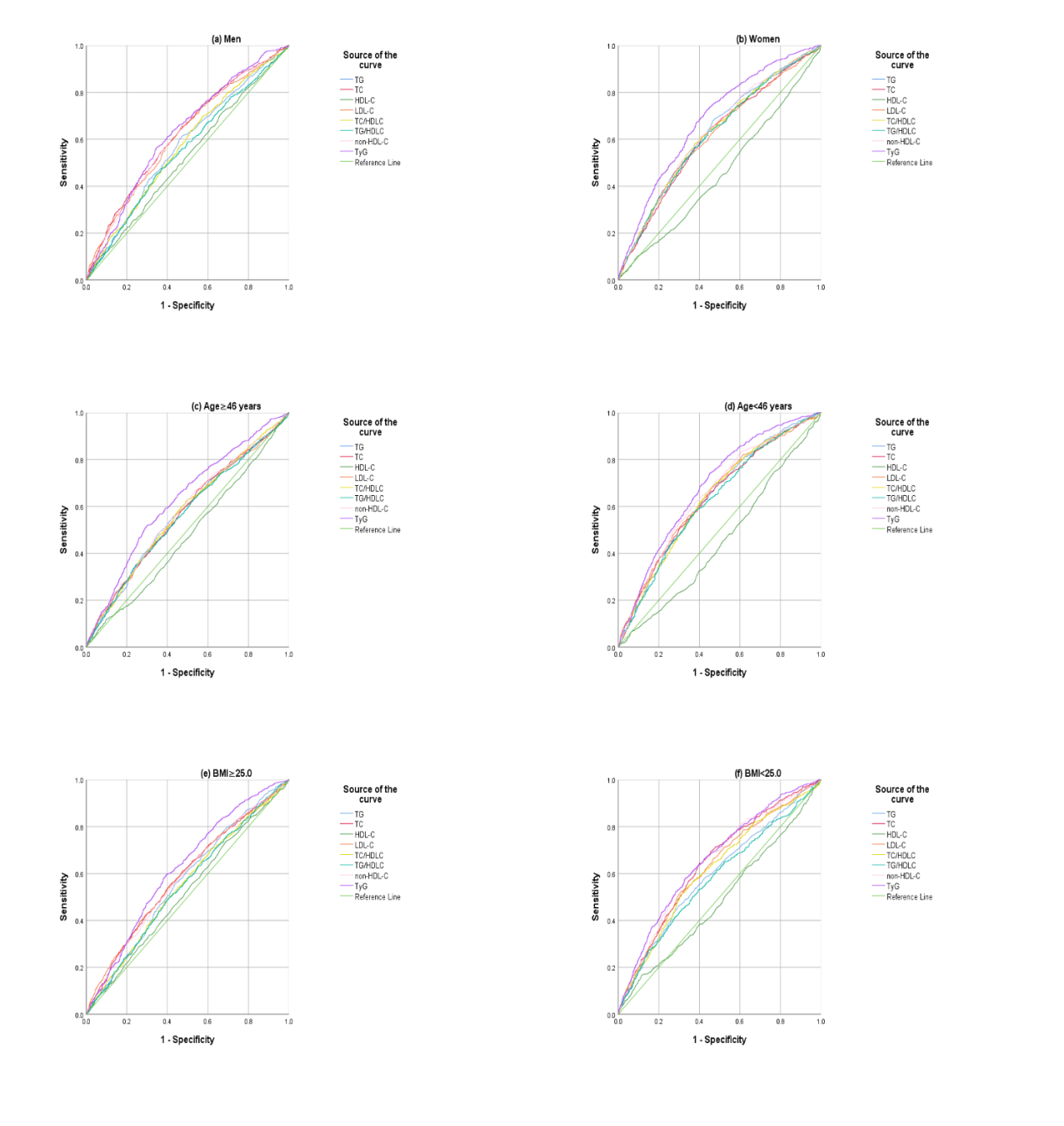
**

Supplement: Supplementary file 9 — Additional file 9: Supplemental Figure 1. ROC curves for predicting prediabetes by lipid index in (a) Men, (b) Women, (c)Age ≥ 46 years, (d) Age < 46 years, (e)BMI ≥ 25.0, and (f) BMI < 25.0. [file 12902_2022_984_MOESM9_ESM.docx]

**
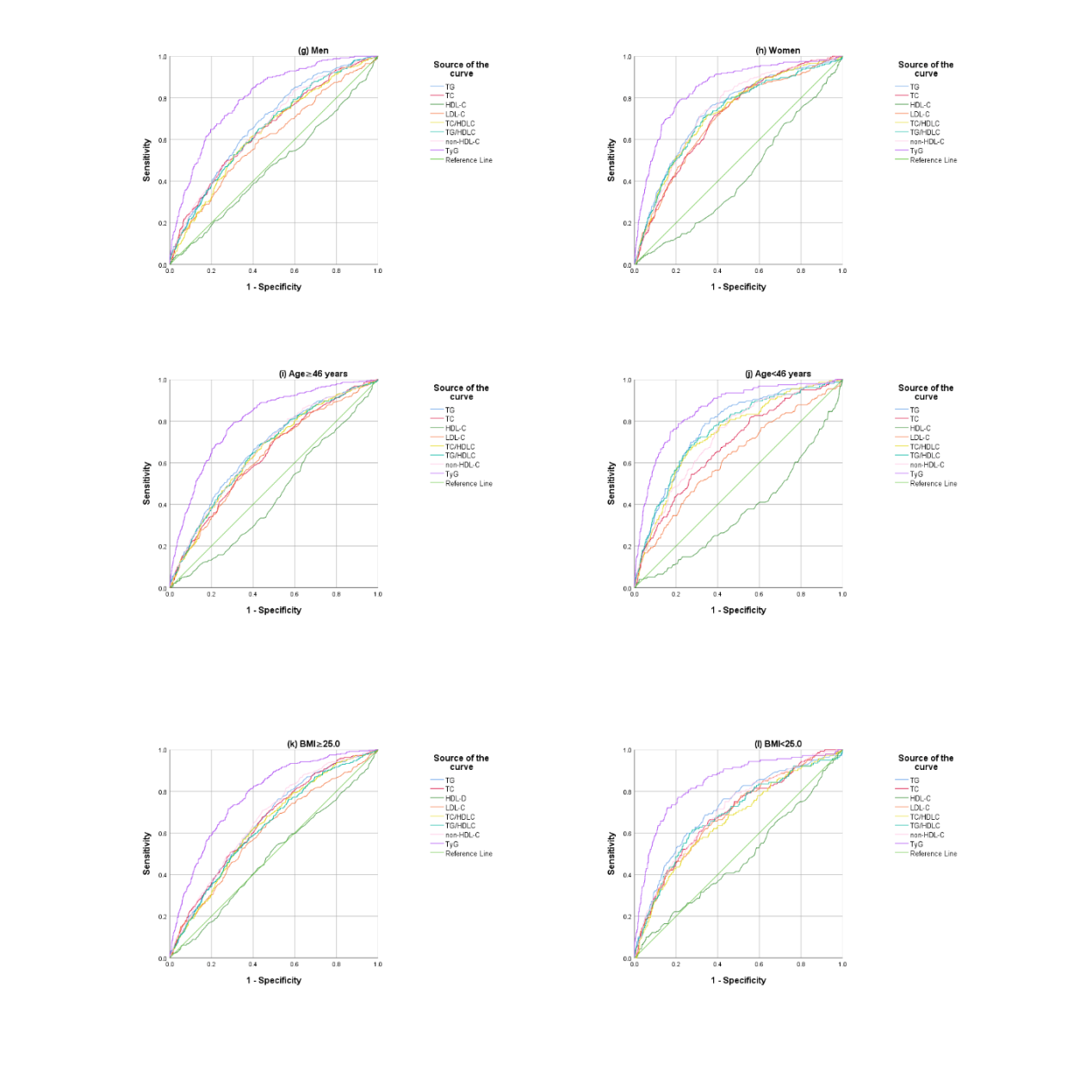
**

Supplement: Supplementary file 10 — Additional file 10: Supplemental Figure 2. ROC curves for predicting diabetes by lipid index in (g) Men, (h) Women, (i)Age ≥ 46 years, (j) Age < 46 years, (k)BMI ≥ 25.0, and (l) BMI < 25.0. [file 12902_2022_984_MOESM10_ESM.docx]
